# KSP: an integrated method for predicting catalyzing kinases of phosphorylation sites in proteins

**DOI:** 10.1186/s12864-020-06895-2

**Published:** 2020-08-04

**Authors:** Hongli Ma, Guojun Li, Zhengchang Su

**Affiliations:** 1 Research Center for Mathematics and Interdisciplinary Sciences, Shandong University, Qingdao, 266237 China; 2 School of Mathematics, Shandong University, Jinan, 250100 China; 3 Department of Bioinformatics and Genomics, The University of North Carolina at Charlotte, Charlotte, NC 28223 USA

**Keywords:** Kinase, Phosphorylation, Kinase-substrate relationship, Algorithm

## Abstract

**Background:**

Protein phosphorylation by kinases plays crucial roles in various biological processes including signal transduction and tumorigenesis, thus a better understanding of protein phosphorylation events in cells is fundamental for studying protein functions and designing drugs to treat diseases caused by the malfunction of phosphorylation. Although a large number of phosphorylation sites in proteins have been identified using high-throughput phosphoproteomic technologies, their specific catalyzing kinases remain largely unknown. Therefore, computational methods are urgently needed to predict the kinases that catalyze the phosphorylation of these sites.

**Results:**

We developed KSP, a new algorithm for predicting catalyzing kinases for experimentally identified phosphorylation sites in human proteins. KSP constructs a network based on known protein-protein interactions and kinase-substrate relationships. Based on the network, it computes an affinity score between a phosphorylation site and kinases, and returns the top-ranked kinases of the score as candidate catalyzing kinases. When tested on known kinase-substrate pairs, KSP outperforms existing methods including NetworKIN, iGPS, and PKIS.

**Conclusions:**

We developed a novel accurate tool for predicting catalyzing kinases of known phosphorylation sites. It can work as a complementary network approach for sequence-based phosphorylation site predictors.

## Background

As a molecular switch in cellular biochemistry, protein phosphorylation by kinases is one of the most ubiquitous post-translational modifications (PTM). It has been estimated that biological activities of 1/3 ~ 2/3 of the proteome of an organism could be regulated by protein phosphorylation [1]. Since protein phosphorylation plays important roles in various biological processes, aberrances of phosphorylation systems are frequently related to various diseases including cancer. Over the past decade, with rapid advancement of high-throughput techniques, a large number of phosphorylation residual sites have been identified and deposited in databases such as PhosphoSitePlus [2], Phospho.ELM [3], and HPRD [4, 5], providing good resources for researchers to investigate the roles of phosphorylation in functional networks of cells. However, for the majority of these phosphorylation sites (p-sites), the cognate catalyzing kinases remain unknown. For example, Phospho.ELM currently comprises 42,914 non-redundant serine, threonine, and tyrosine p-sites in more than 11,000 protein sequences, but only ∼12% of these sites have annotated cognate kinases. On the other hand, kinases comprise the putative targets of about 20% drugs on the market [6], however, most of their substrate sites are unknown. Clearly, prediction of the substrate sites of kinases can help elucidate underlying mechanisms. Therefore, it is imperative to develop new methods to predict catalyzing kinases for the exponentially increasing number of p-sites in proteins, thereby revealing targets of therapeutics [7–9].

Indeed, many computational methods have been developed to address the demand. These methods can be divided into two categories. The sequence-based methods only use flanking sequence around a p-site to predict the catalyzing kinases [10–14]; while the combined methods integrate flanking sequences around a p-site with other types of data, such as protein disorder regions, sequence similarity between kinase families, and protein-protein interactions (PPI) to predict the catalyzing kinases [11, 15–20]. Among the existing network methods, most were developed based on the similarity between sequences [8, 11, 15, 21, 22], and do not use topological information of known interaction networks [8, 22, 23]. To overcome this shortage, KSIBW adopted a new edge clustering coefficient (NECC) to refine the weight of PPI networks [21]. However, there remains room of improvement to accurately capture the similarity between nodes in PPI networks [21, 24].

In this study, we propose a novel combined method, termed KSP, to predict kinases of given p-sites in proteins. Firstly, we constructed an interaction network by integrating known kinase-substrate relationships and known PPI. Secondly, we converted the interaction network into a bipartite graph consisting of two types of nodes: kinases and non-kinase proteins, and then assigned to each edge of the bipartite graph a weight computed by a newly designed similarity score. In addition, we provided complementary sequence-based scoring methods named PWMScore (Position Weight Matrix Score) and CBS (Clustering for BLOSUM62 similarity). Therefore, a user can perform both network-based and combined predictions. When tested on several p-sites with known kinases, KSP was able to accurately predict cognate kinases for the p-sites. KSP also outperformed NetworKIN, iGPS, PKIS, and sequence-alone methods on the datasets measured by the ROC (receiver operating characteristic) curve, the F1 score (harmonic average of the precision and recall) and the PRC (precision-recall curve).

## Results

### Predicting kinase-substrate relationships

We first performed 10-fold cross-validation to evaluate KSP on all kinase-substrate interaction pairs. If the true kinase for a substrate protein was included in the top 10 kinases ranked by KSPScore, we count it as a true prediction. The accuracy of the prediction is defined as the ratio of the number of true predictions to the size of test dataset. Eventually, we reached accuracies 82.9%, 84.7%, 82.8%, 85.2%, 85.9%, 83.0%, 84.1%, 84.4%, 86.5%, 85.1% respectively on each fold.

**Fig. 1:**
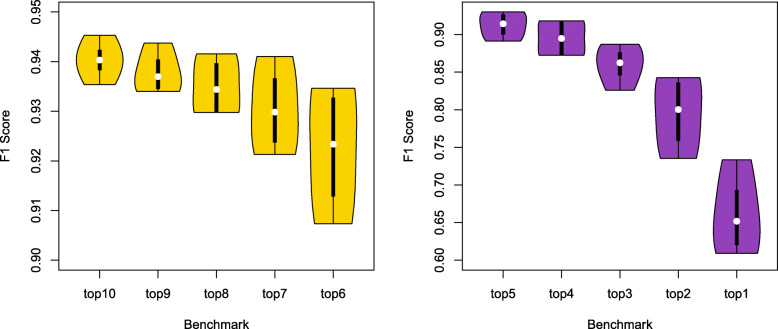
Vioplot of F1 Scores of the predictions in the four kinases as a whole when different number of top predictions were considered

To evaluate the performance of KSP for specific kinases, we randomly divided the p-sites of a kinase into a training set and a positive test set using a ratio of 7:3. The negative test set contains both p-sites of other kinases and the sequences around S/T/Y residues without known phosphorylation. The test set is formed by the positive test set and the negative test set with a ratio of 1:1. Table 1 shows the results of predicting kinases for the p-sites of CK2A1 and Src when the top 1, 2, …, and 10 ranked kinases were considered. The results of PKACA and CDK1 are listed in Additional file 2. The F1 score is the harmonic average of the precision and recall that is a measure of a test’s accuracy. As expected, the accuracy of the prediction decreases as the benchmark becomes stricter (Fig. 1).

**Table 1:** Evaluation of KSP on CK2A1 and Src when different number of top-ranked predictions were considered

**kinase: CK2A1**	top 10	top 9	top 8	top 7	top 6	top 5	top 4	top 3	top 2	top 1
TP	458	453	451	447	436	426	415	389	351	274
FP	46	42	42	38	32	30	24	23	17	8
TN	319	323	323	327	333	335	341	342	348	357
FN	7	12	14	18	29	39	50	76	114	191
TPR	0.984946	0.974194	0.969892	0.961290	0.937634	0.916129	0.892473	0.836559	0.754839	0.589247
FPR	0.126027	0.115068	0.115068	0.104110	0.087671	0.082192	0.065753	0.063014	0.046575	0.021918
TNR	0.873973	0.884932	0.884932	0.895890	0.912329	0.917808	0.934247	0.936986	0.953425	0.978082
FNR	0.015054	0.025806	0.030108	0.038710	0.062366	0.083871	0.107527	0.163441	0.245161	0.410753
ACCURACY	0.936145	0.934940	0.932530	0.932530	0.926506	0.916867	0.910843	0.880723	0.842169	0.760241
PRECISION	0.908730	0.915152	0.914807	0.921649	0.931624	0.934211	0.945330	0.944175	0.953804	0.971631
RECALL	0.984946	0.974194	0.969892	0.961290	0.937634	0.916129	0.892473	0.836559	0.754839	0.589247
F1	0.945304	0.943750	0.941545	0.941053	0.934620	0.925081	0.918142	0.887115	0.842737	0.733601
**kinase: Src**	top 10	top 9	top 8	top 7	top 6	top 5	top 4	top 3	top 2	top 1
TP	395	394	392	389	384	372	360	328	300	214
FP	38	37	35	35	32	20	18	16	12	8
TN	331	332	334	334	337	349	351	353	357	361
FN	13	14	16	19	24	36	48	80	108	194
TPR	0.968137	0.965686	0.960784	0.953431	0.941176	0.911765	0.882353	0.803922	0.735294	0.524510
FPR	0.102981	0.100271	0.094851	0.094851	0.086721	0.054201	0.048780	0.043360	0.032520	0.021680
TNR	0.897019	0.899729	0.905149	0.905149	0.913279	0.945799	0.951220	0.956640	0.967480	0.978320
FNR	0.031863	0.034314	0.039216	0.046569	0.058824	0.088235	0.117647	0.196078	0.264706	0.475490
ACCURACY	0.934363	0.934363	0.934363	0.930502	0.927928	0.927928	0.915058	0.876448	0.845560	0.740026
PRECISION	0.912240	0.914153	0.918033	0.917453	0.923077	0.948980	0.952381	0.953488	0.961538	0.963964
RECALL	0.968137	0.965686	0.960784	0.953431	0.941176	0.911765	0.882353	0.803922	0.735294	0.524510
F1	0.939358	0.939213	0.938922	0.935096	0.932039	0.930000	0.916031	0.872340	0.833333	0.679365

### Improving sequence-based prediction of kinase-substrate relationship

**Fig. 2:**
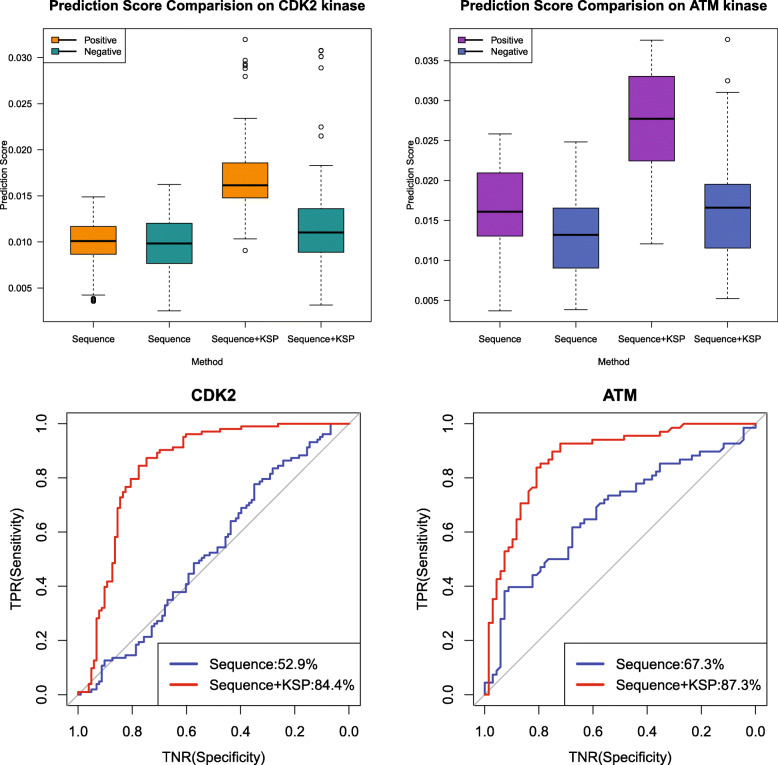
Comparison between the sequence-based method and the combined method (Sequence + KSP) on the p-sites of CDK2 and ATM. The two boxplots on the top show the different precision score of positive and negative samples of the sequence-based method and the combined method. The two figures on the bottom show the ROC curves of these two methods on the p-sites of CDK2 and ATM

We captured the frequency and similarity features of local sequences around p-sites using PWMScore and CBSScore. In order to validate the performance of KSP in improving sequence-based prediction methods (PWM and CBS), we defined SequenceScore as the sum of normalized PWMScore and normalized CBSScore, and the OverallScore as the sum of normalized KSPScore and SequenceScore. Moreover, as kinases of the same families have very similar p-sites with similar flanking local sequences, to fully evaluate the sensitivity of KSP, we generated a test dataset in which the negative samples were from the substrates of the same kinase families of CDK2 and ATM (see Additional file 3, Additional file 4, and Additional file 8). As shown in Fig. 2, the difference of OverallScores between positives and negatives is much larger than that of their respective SequenceScores. Thus, KSP largely improved the sequence-based methods with its ability to distinguish between positives and negatives more efficiently. When adding KSP, the ROC curves show a remarkable increase in the AUROC (the area under the ROC curve) values: CDK2 by 31.5% and ATM by 20% (Fig. 2).

**Fig. 3:**
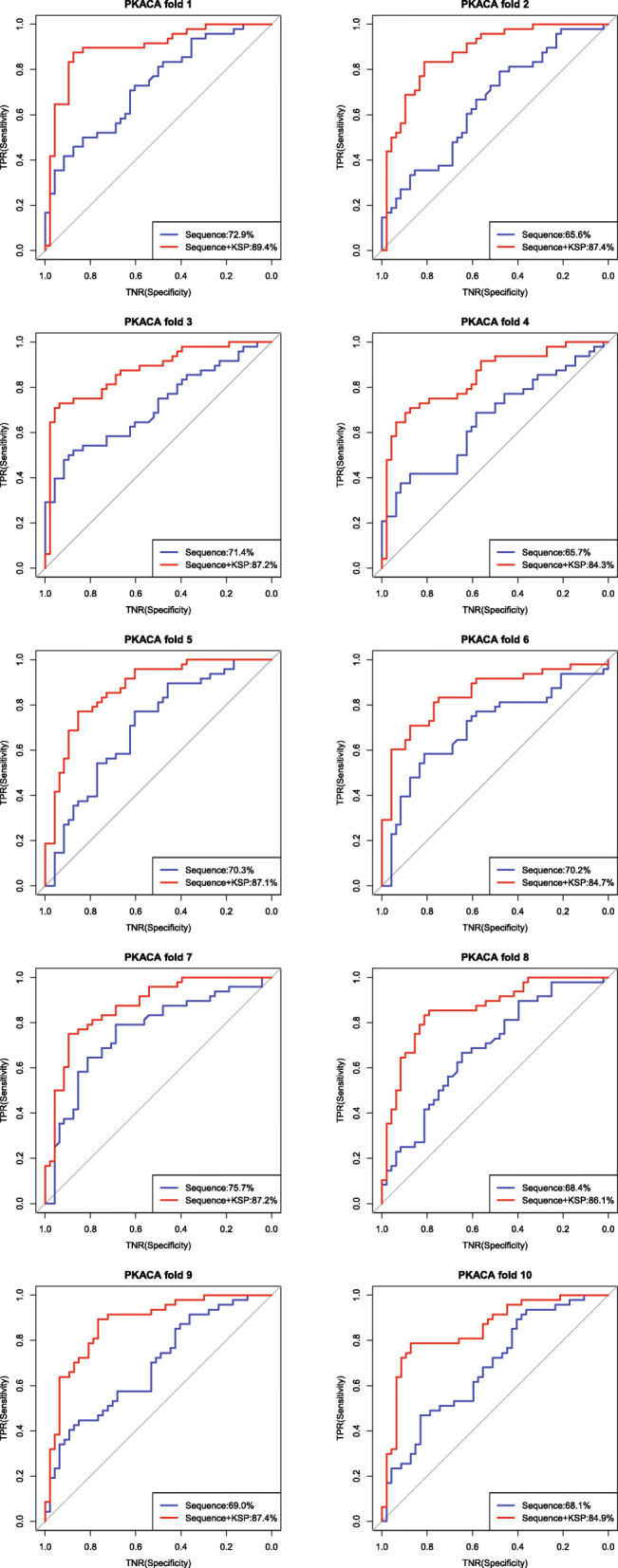
Results of the 10-fold cross validation on PKACA: the ROC curves of the sequence-based method and the combined method (Sequence+KSP)

In addition, we also compared the performance of these methods on two kinases (PKACA and PKCA) by using 10-fold cross-validation. As shown in Fig. 3, KSP significantly improved the sequence-based method in terms of the AUROC values on PKACA. Similar results were seen for PKCA (Additional file 9).

### Comparison with alternative methods

Finally, we compared KSP, PWMScore and CBS with the state-of-the-art methods NetworKIN [9, 23] and iGPS [25] on substrates of two kinases (CDK2 and ATM), using the same training set and test set as described above. To run iGPS on the data, we modified the format of flanking sequence of p-sites according to the requirement of iGPS and selected all-score output rather than a threshold. Besides, because NetworKIN does not use p-sites as the input in the form of 15-mers as in PhosphoSitePlus, we tested it by inputting substrate protein sequences and positions of p-sites. The Minimum score of output was set to be 0 with Max difference set to be the default value. The details of the output scores can be found in Additional file 3 and Additional file 4.

**Fig. 4:**
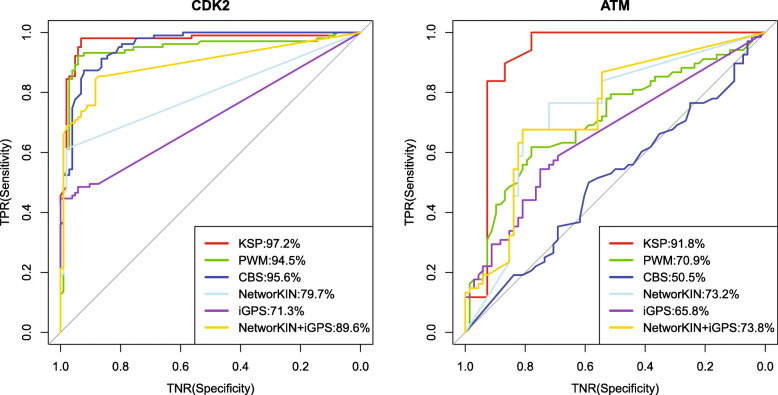
Comparison between KSP and five other methods (including one combined method) on CDK2 and ATM

As shown in Fig. 4, KSP has the most accurate prediction on the two kinases compared to other methods. Sequence-based methods including PWMScore and CBS are not robust enough for different kinases, their performance may depend on the size of validated p-sites and the choice of negatives in the test set. Although the combination of NetworKIN and iGPS improves AUROC significantly on CDK2 and slightly on ATM, it still cannot match the precision of KSP.

**Fig. 5:**
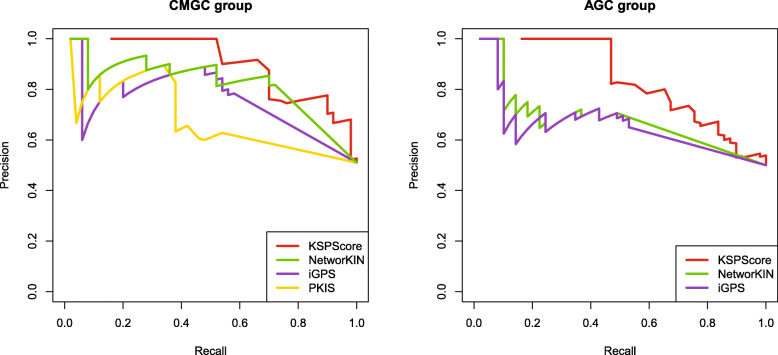
Comparison between KSP and other methods on the CMGC and AGC groups

Besides, for a fairer comparison, we reconstructed our similarity bipartite graph B using the kinase-substrate pairs collected from Phospho.ELM, which were used by NetworKIN, iGPS and PKIS as well. After removing redundant and missing data, we found that the number of known p-sites of each kinase is too small, so we only trained and tested on two kinase groups (CMGC group and AGC group). The precision-recall curves of the predictions in Fig. 5 show that KSPScore also outperformed NetworKIN and iGPS on the CMGC group and the AGC group. Here, we only tested the CMGC group on PKIS and compared it with other tools because it could not provide predictions for AGC groups.

## Discussion

Phosphorylation of proteins by kinases plays a crucial role in protein functions in cells [26]. It is estimated that human genome encodes 518 protein kinase genes, comprising 134 families. These kinases are responsible for almost all of the human protein phosphorylation events, which are involved with various biological processes [27]. Therefore, it is of importance to figure out the kinase-substrate relationships in order to understand the molecular mechanisms underlying these biological processes and construct phosphorylation networks [28]. Although an increasing number of p-sites have been identified using high throughput methods, finding their cognate kinases has become a bottleneck. Here, we show that a network scoring tool KSP which integrates kinase-substrate relationships and PPI is able to accurately predict cognate kinases of p-site substrates. Furthermore, our scoring function (KSPScore) can better capture similarities of kinases than commonly used similarity indices. The aim of KSP is to predict candidate catalyzing kinases of the numerous experimentally identified p-sites, and it can be used as an assistant tool for other kinase phosphorylation prediction software. Meanwhile, we also provided two sequence-based methods (PWMScore and CBSScore) to predict the kinase of a query p-site using amino acids frequencies and BLOSUM 62 similarity, respectively, and we showed that the PWM and CBS methods could make better use of known local kinase-specific conserved sequences to predict kinase-substrate relationships for many kinases. Compared with the existing well-regarded methods (e.g., iGPS and NetworKIN) [9, 23, 25], KSP presents fairly robust high-performance in terms of the accuracy on several kinases and kinase groups.

Although some substrate-kinase relationship predictors considered other types of information like protein disorder regions [29] as well as cell cycles, the inclusion of these kinds of information seems to have little improvement for most kinases [11, 16, 18, 20, 30]. Thus, we only consider two well-recognized features, protein interactions and conserved local sequence around phosphorylation sites. Since genetic variation changes phosphorylation sites or their interacting kinases [31, 32], many methods have emerged to quantify the effects of SNVs (single nucleotide variants) on protein phosphorylation. ActiveDriver identified a specific p-site region in a given protein that has a significantly different mutation rate than expected, thereby finding cancer driver mutations [33, 34]. MIMP and PhosphoPICK-SNP provided tools to predict loss or gain of protein phosphorylation sites based on methods of predicting p-sites [12, 35]. After constructing this powerful interaction network, the next step for us is to utilize this tool to predict the impact of mutations on substrate-kinase relationships. Furthermore, one potential concern is that our prediction only works well on a few kinases due to the unbalanced distribution of kinase information, with the availability of more data of phosphorylation and protein interactions in the future, the scope of application as well as the accuracy of our methods can be further improved.

## Conclusions

In this paper, we developed a novel method, KSP, to predict catalyzing kinases of query p-sites in proteins. This method is based on the connection relationship in a combined phosphorylation network and outperforms existing kinase-substrate relationship prediction tools on multiple datasets. We believe that KSP will aid in the efforts to elucidate the protein kinase regulation mechanisms, especially for the kinases that have not been well studied.

## Methods

### Data collection and preprocessing

Experimentally verified human p-sites with kinase information and sequences were downloaded from the latest PhosphoSitePlus [2] and Phospho.ELM [3]. After removing the redundant and missing data, we collected 10,198 known human kinase-substrate pairs for 370 kinases. The detailed information of these kinases was summarized in Additional file 1. In order to understand the structure of the kinase-substrate interaction network, we visualized it with Cytoscape [36] (see Additional file 5) and found that the network is heterogeneous, that is, a small number of nodes in the network have very large numbers of connections, while most nodes have very few connections [37, 38] (see Additional file 6). We then constructed a new network by integrating the kinase-substrate interaction network and the human PPI network extracted from HPRD (Human Protein Reference Database) [4, 5] by taking the union of their nodes and edges, and deleting all the components but the largest one. The integrated network consists of two types of nodes: those representing the kinases; and those representing other proteins. We then conducted a statistical analysis of the degree distribution of nodes in this integrated network (see Additional file 7).

We retained ± 7 flanking residues of p-sites of different kinases to capture local sequence features, and only selected those kinases with greater than 15 p-sites. After removing duplicates for each kinase using CD-HIT [39], we ended up with 113 kinases.

### Kinase-substrate prediction score (KSPScore)

**Fig. 6:**
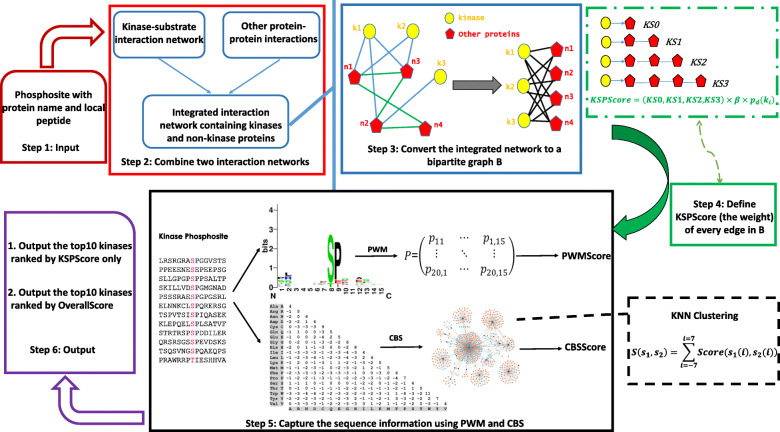
Flowchart of KSP for kinase-substrate pair prediction

In a complex network, there are many indices between two different nodes, including similarity indices, matching indices and statistic-based indices [40]. In this study (Fig. 6), we constructed a complex network *G* = (*V*, *E*_1_) by combining the kinase-substrate network and the PPI network, where nodes represent kinases and other proteins, and edges represent catalytic relationships between kinases and proteins of substrate p-sites and interactions among the remaining proteins. Networks are reduced to protein interaction levels by removing the p-sites information. By *w*_*i*, *j*_, we denote the weight of the edge between nodes *i* and *j* in *G* according to the number of identified interactions.

For a given kinase *k* and a p-site *p* in a substrate protein *n*, we consider all neighbors of *k* and *n* in *G* = (*V*, *E*_1_). Let *N*_*x*_ (*x* ∈ *V*) be the set of neighbors of *x*, *d*_*x*_ (*x* ∈ *V*) be the degree of *x*, and *Z*_*i*, *j*_ (*i*, *j* ∈ *V*) be the set of common neighbors of *i* and *j*. We calculate the similarity score *KSPScore* (*k*, *n*) between *k* and *n* as follows.

1. If *n* ∈ *N*_*k*_ ∩ *V,*
  $$ KS0={w}_{k,n} $$
2. Besides, if *Z*_*k*, *n*_ ≠ ø,
  $$ KS1=\sum \limits_{v_k\in {Z}_{k,n}}{w}_{k,{v}_k}{w}_{v_k,n} $$
3. Besides, if ${Z}_{v_p,n}\ne \varnothing$ when *v*_*p*_ ∈ *N*_*k*_ ∩ *V*,
  $$ KS2=\sum \limits_{v_p\in {N}_k}\sum \limits_{v_q\in {Z}_{v_p,n}}{w}_{k,{v}_p}{w}_{v_p,{v}_q}{w}_{v_q,n} $$
4. Besides, if ${Z}_{v_x,{v}_y}\ne \varnothing$ when *v*_*x*_ ∈ *N*_*k*_ ∩ *V*, *v*_*y*_ ∈ *N*_*n*_ ∩ *V*,
  $$ KS3=\sum \limits_{v_x\operatorname{}{N}_k}\sum \limits_{v_y\operatorname{}{N}_n}\sum \limits_{v_k\operatorname{}{Z}_{v_x,{v}_y}}{w}_{k,{v}_x}{w}_{v_x,{v}_k}{w}_{v_k,{v}_y}{w}_{v_y,n} $$

Finally, $$ KSPScore\left(k,n\right)=\left( KS0, KS1, KS2,\operatorname{}\operatorname{} KS3\right)\times \beta \times {p}_d(k) $$

*β* = (*β*_0,_ *β*_1,_ *β*_2_, *β*_3_)^*T*^ is a parameter vector of components *β*_0_, *β*_1_, *β*_2_, *β*_3_ which add up to 1 and *p*_*d*_ is the punitive function:

$$ {p}_d(x)=\left\{\begin{array}{c}1-\frac{\left({\mathit{\log}}_2{d}_x-{\mathit{\min}}_{i\in V}\left({\mathit{\log}}_2{d}_i\right)\right)\times 0.2}{{\mathit{\max}}_{i\in V}\left({\mathit{\log}}_2{d}_i\right)-{\mathit{\min}}_{i\in V}\left({\mathit{\log}}_2{d}_i\right)},{d}_x>2\ \\ {}1,{d}_x\le 2\end{array}\right. $$

We define the *KSPScore* (*k*, *n*) based on the assumption that correlation between two nodes in a biological network can be further supported by interactions among their neighbors. In order to reduce the bias to over-studied kinases, we diminish impact of the interaction between a kinase and a substrate protein by using the punitive function *p*_*d*_ and adjusting parameter *β* in the *KSPScore* formula. By default, we set *β* = (0.25, 0.225, 0.1875, 0.1875)^*T*^.

Next, we convert *G* into a weighted bipartite graph *B* = (*K*, *N*, *E*_2_, *W*), where *K* ∪ *N* = *V*, with *K* representing kinases, *N* non-kinase proteins, *E*_2_ the edges between *K* and *N*, and *W* the weights on *E*_2_ defined as the *KSPScore* s. Here we only connect a kinase and a non-kinase protein if their *KSPScore* ≠ 0. We define the KSPScore between kinase *k*_*i*_ and a p-site *p* as *KSPScore*(*k*_*i*_, *p*) = *KSPScore*(*k*_*i*_, *n*), where *i* = 1, 2, …, 370 and *n* represent the substrate protein of *p*. For a query p-site we consider all the 370 kinases, and output 10 top-ranked kinases as possible cognate candidates.

### Position weight matrix score (PWMScore)

We modeled sequence specificity of the p-sites of a kinase using a position weight matrix (PWM) following the method of MIMP [12] and constructed PWMs for 113 kinases with more than 15 p-sites. To construct a PWM, we first generated a position frequency matrix (PFM) by counting the occurrences of each amino acid at each position in the multiple alignments of the p-sites of length L, and the position profile matrix (PPM) by dividing the PFM by N, the number of p-sites. Finally, the PWMs were calculated by taking log likelihoods. Formally, let *X* be a set of *N* p-sites’ sequences of length L = 15, and *M* = (*M*_*k*, *j*_) the PWM of *X,* then the elements *M*_*k*, *j*_ of the PWM were calculated by

$$ {M}_{k,j}={\log}_2\left(\frac{1}{N{b}_k}\sum \limits_{i=1}^NI\left({X}_{i,j}=k\right)\right) $$

where *i* = 1, …, *N*; *j* = 1, …, 15; *k* is one of amino acids; and *I*(*a* = *k*) is an indicator function where *I*(*a* = *k*)= 1 if *a* = *k* and 0 otherwise; *b*_*k*_ is the background frequency of amino acid *k*.

For a query p-site *p* and kinase *k*_*i*_, we defined *PWMScore*(*k*_*i*_, *p*) as the sum of the relevant values at each position in the PWM of *k*_*i*_, where *i* = 1, 2, …, 113. For a query p-site we consider all the 113 PWMs, and output 10 top-ranked kinases as cognate candidates.

### Clustering for BLOSUM62 similarity (CBS)

Flanking sequences around the p-sites of a kinase often show some similarity, to use this feature for predicting kinase-substrate relationships, we propose a KNN (k-nearest neighbors) based clustering method. We define the similarity score *S* between sequences *s*_1_ and *s*_2_ as

$$ S\left({s}_1,{s}_2\right)={\sum}_{i=-7}^{i=7} Score\left({s}_1(i),{s}_2(i)\right), $$

where *Score*(*a*, *b*) is the alignment score between amino acids a and b according to an amino acid substitution matrix [41] (BLOSUM62 by default), and it is defined to be 0 if the upstream or downstream regions of the sites have less than 7 residues. For each p-site sequence *s*_*j*_ (15-mers including ± 7 flanking residues around the phosphorylated amino acid), we find its *k* nearest neighbors in the training set according to similarity score *S* (The larger the similarity score between two sequences, the closer they are). We then calculate the *CBSScore*(*k*_*i*_, *s*_*j*_) between kinase *k*_*i*_ and sequence *s*_*j*_ as the percentage of the sites catalyzed by *k*_*i*_ in the *k* nearest neighbors of *s*_*j*_.

For the input local sequence *s*_*j*_ of a query p-site, we consider all the 113 kinases, and output 10 top-ranked kinases as possible cognate candidates. We tested different k (1%, 2.5%, 5% and 7.5% of the size of the whole training dataset) for the kinase prediction and finally set the default k to be 7.5% of the size of the training dataset.

## Supplementary information

**Additional file 1: Table S1.** Kinases information collected in KSP.

**Additional file 2: Table S2.** Evaluation of KSP on PKACA and CDK1 when different number of top-ranked predictions were considered.

**Additional file 3: Table S3.** test set of ATM kinase including peptides, interactions, and output scores of all softwares.

**Additional file 4: Table S4.** test set of CDK2 kinase including peptides, interactions, and output scores of all softwares.

**Additional file 5: Figure S1.** Visualized kinase-substrate interaction network.

**Additional file 6: Figure S2.** The distribution of kinase information validated in PhosphoSitePlus dataset of 370 kinases.

**Additional file 7: Figure S3.** The degree distribution of kinase and other non-kinase proteins in the intergrated network.

**Additional file 8: Figure S3.** The frequency heatmap of the positive samples and negative samples of ATM kinase.

**Additional file 9: Figure S5.** Results of the 10-fold cross validation experiment on PKCA.

## Data Availability

All the dataset and source code, which can be used to test this method, are available at https://sourceforge.net/projects/kspscore/files/

## Abbreviations

PTM: Post-translational modification
p-sites: phosphorylation sites
PPI: Protein-protein interactions
NECC: New edge clustering coefficient
PWMScore: Position weight matrix score
CBS: Clustering for BLOSUM62 similarity
ROC: Receiver operating characteristic
PRC: Precision-recall curve
HPRD: Human Protein Reference Database
KSPScore: Kinase-Substrate Prediction Score
PWM: Position weight matrix
PFM: Position frequency matrix
PPM: Position profile matrix
KNN: k-nearest neighbors
AUROC: The area under the ROC curve
SNV: Single nucleotide variants
