# Supplementary material for: KSP: an integrated method for predicting catalyzing kinases of phosphorylation sites in proteins
Source: BMC Genomics. 2020 Aug 4;21:537. doi: 10.1186/s12864-020-06895-2 (PMC7646512; doi:10.1186/s12864-020-06895-2)
Supplement: Supplementary file 5 — Additional file 5: Figure S1. Visualized kinase-substrate interaction network. [file 12864_2020_6895_MOESM5_ESM.pdf]

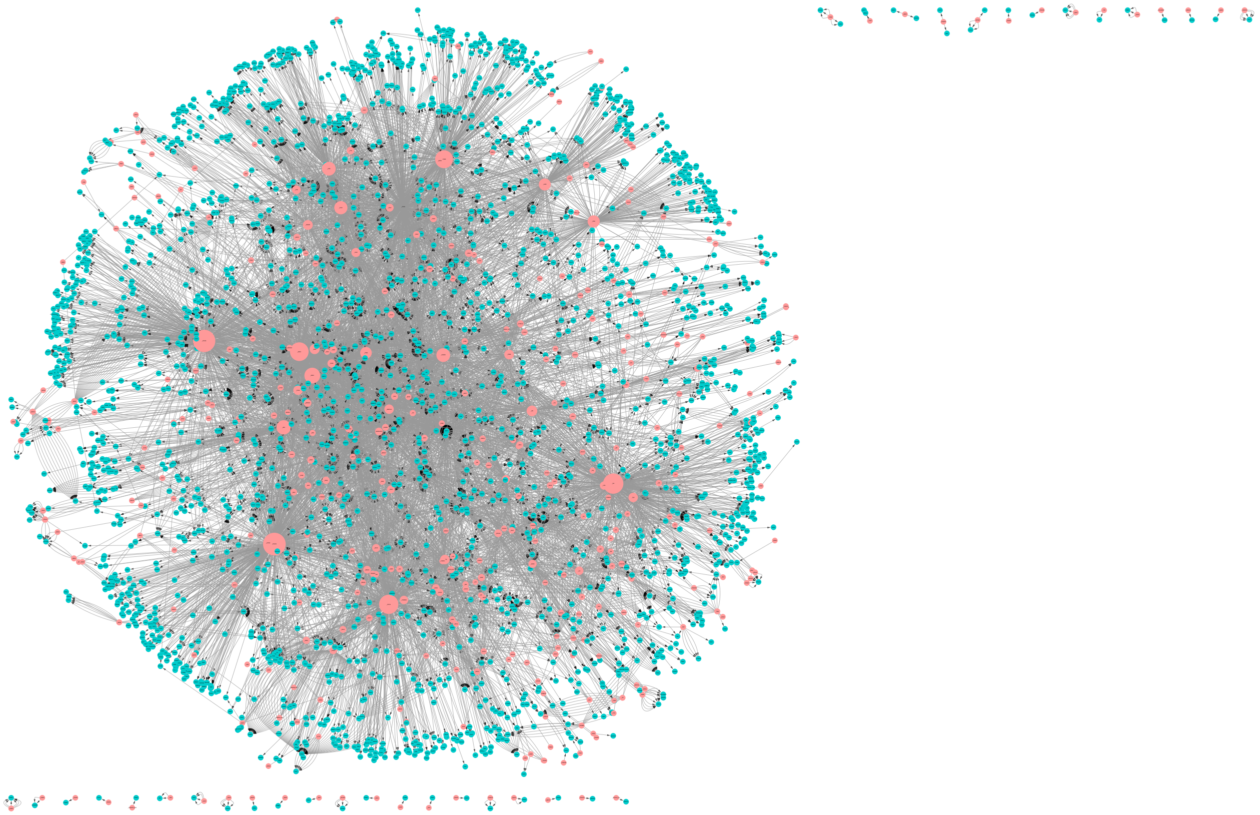

Figure S1. Visualized kinase-substrate interaction network.(best viewed by zooming in a PDF viewer). Red and green circles denote kinases and substrates respectively. An edge corresponds to an identified kinase – substrate interaction. The size of the nodes denote the degree of them. The network is composed of 10369 edges and 2707 nodes (of which 370 are kinases).
