# Supplementary material for: KSP: an integrated method for predicting catalyzing kinases of phosphorylation sites in proteins
Source: BMC Genomics. 2020 Aug 4;21:537. doi: 10.1186/s12864-020-06895-2 (PMC7646512; doi:10.1186/s12864-020-06895-2)
Supplement: Supplementary file 6 — Additional file 6: Figure S2. The distribution of kinase information validated in PhosphoSitePlus dataset of 370 kinases. [file 12864_2020_6895_MOESM6_ESM.pdf]

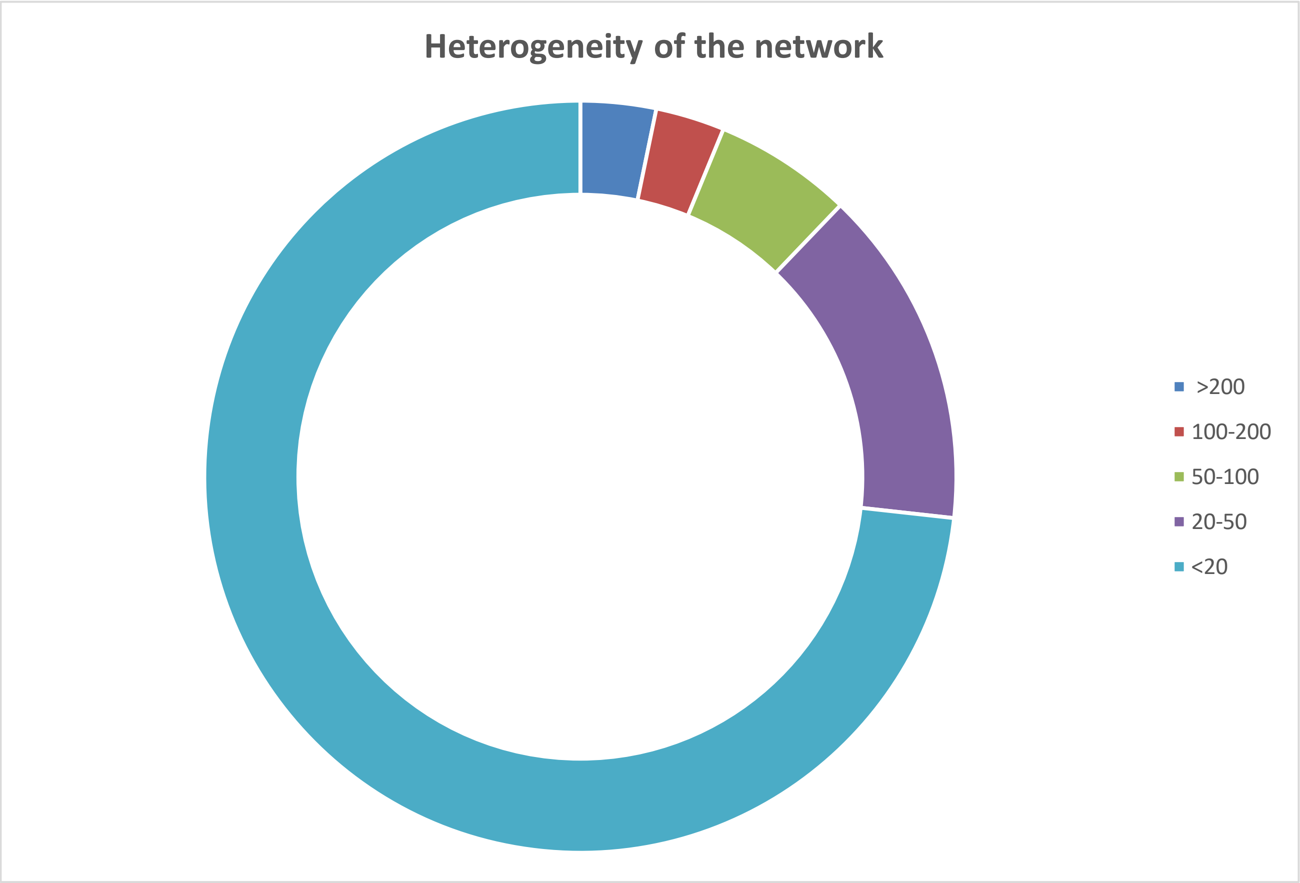

Figure S2. The distribution of kinase information validated in PhosphoSitePlus dataset of 370 kinases.
