# Supplementary material for: KSP: an integrated method for predicting catalyzing kinases of phosphorylation sites in proteins
Source: BMC Genomics. 2020 Aug 4;21:537. doi: 10.1186/s12864-020-06895-2 (PMC7646512; doi:10.1186/s12864-020-06895-2)
Supplement: Supplementary file 7 — Additional file 7: Figure S3. The degree distribution of kinase and other non-kinase proteins in the intergrated network. [file 12864_2020_6895_MOESM7_ESM.pdf]

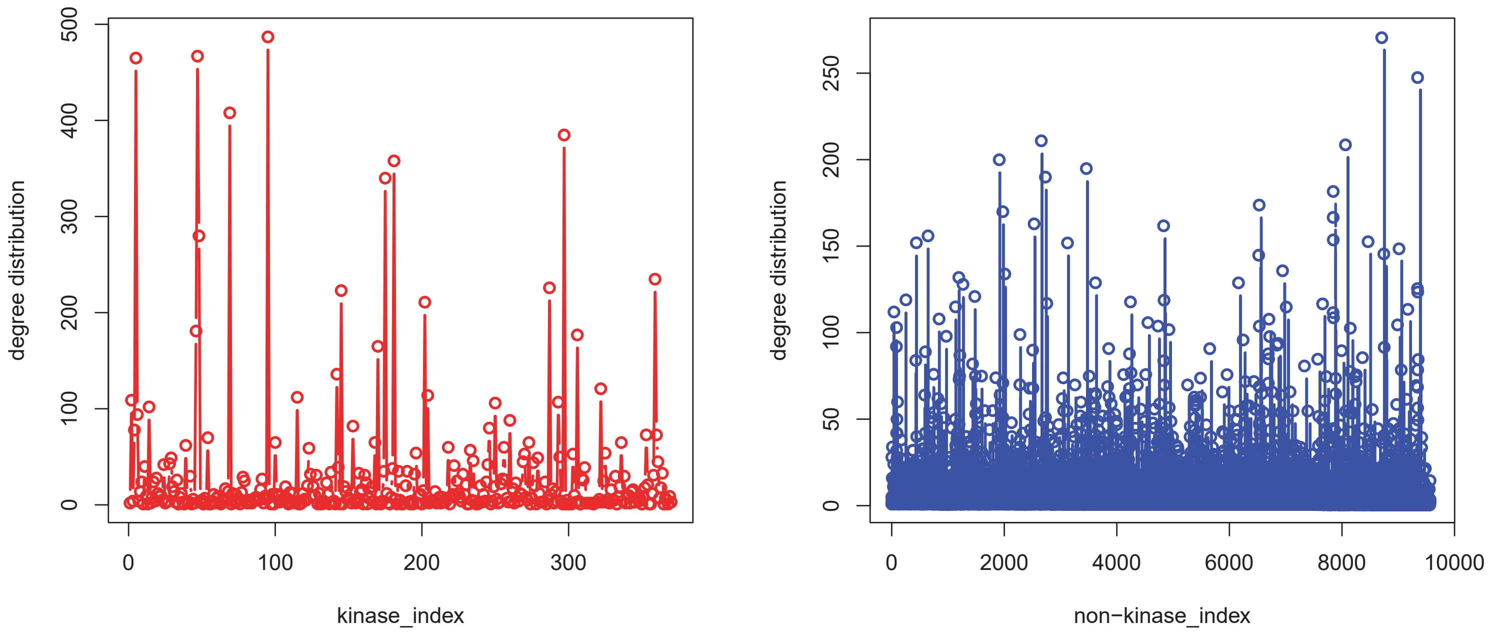

Figure S3. The degree distribution of kinase and other non-kinase proteins in the intergrated network.
