# Supplementary material for: KSP: an integrated method for predicting catalyzing kinases of phosphorylation sites in proteins
Source: BMC Genomics. 2020 Aug 4;21:537. doi: 10.1186/s12864-020-06895-2 (PMC7646512; doi:10.1186/s12864-020-06895-2)
Supplement: Supplementary file 8 — Additional file 8: Figure S3. The frequency heatmap of the positive samples and negative samples of ATM kinase. [file 12864_2020_6895_MOESM8_ESM.pdf]

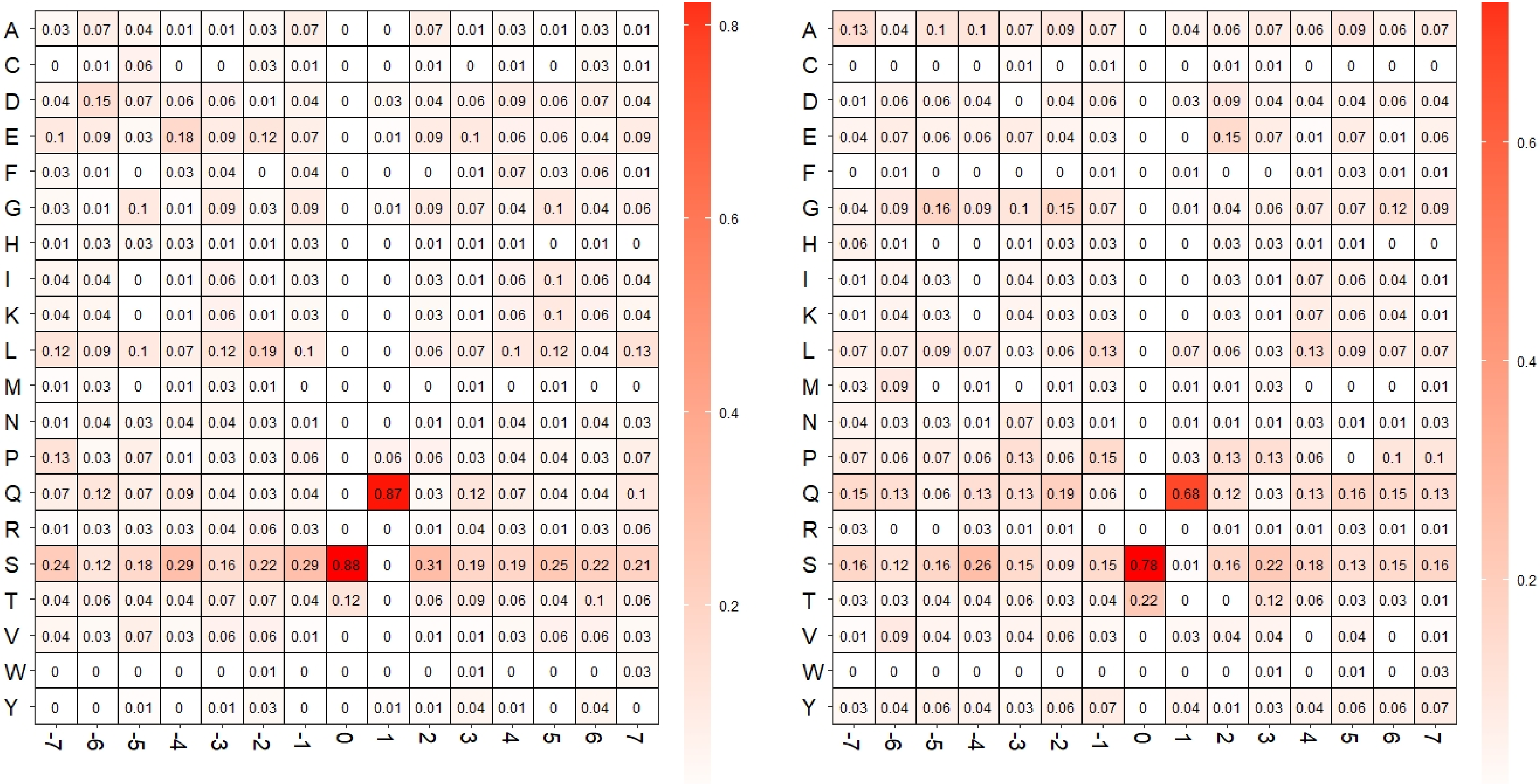

Figure S4. The frequency heatmap of the positive samples and negative samples of ATM kinase. The similar probability distribution of amino acids in each position reveals the similar sequence feature of the positive and negative samples.
