# Supplementary figures and images for: KSP: an integrated method for predicting catalyzing kinases of phosphorylation sites in proteins
Source: BMC Genomics. 2020 Aug 4;21:537. doi: 10.1186/s12864-020-06895-2 (PMC7646512; doi:10.1186/s12864-020-06895-2)

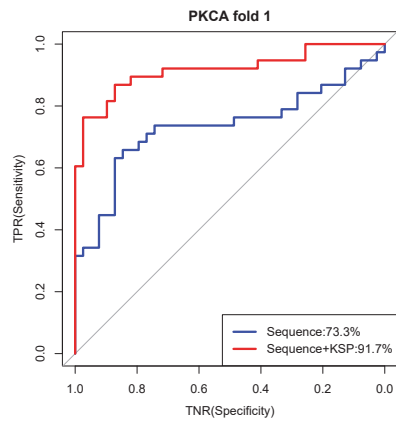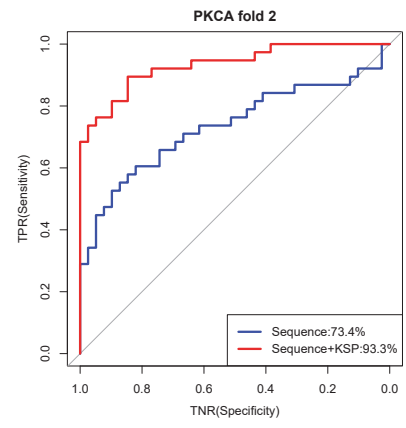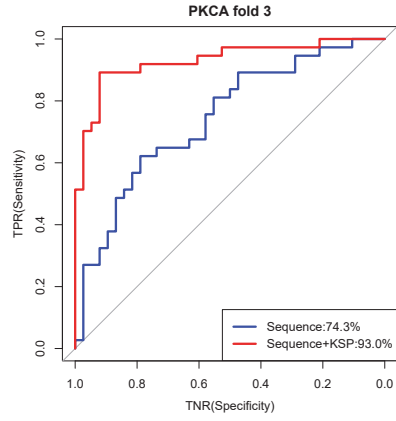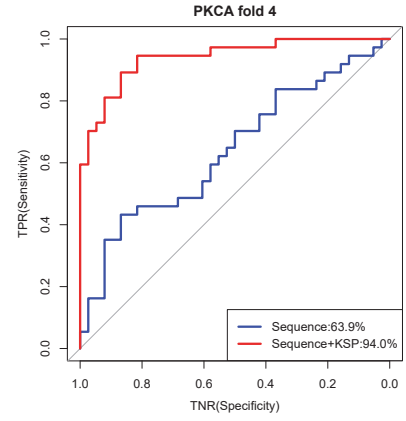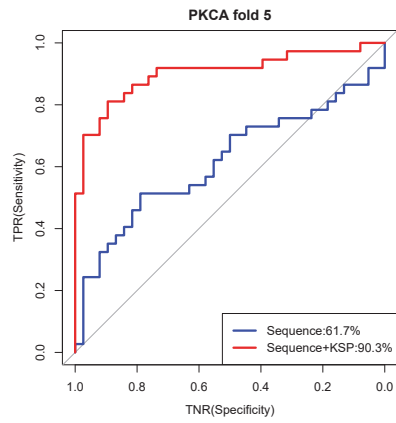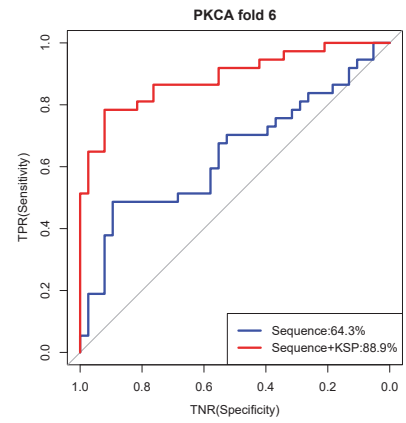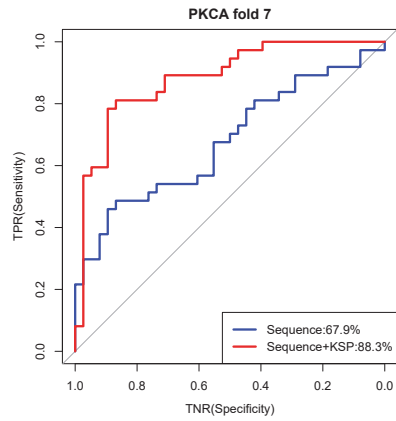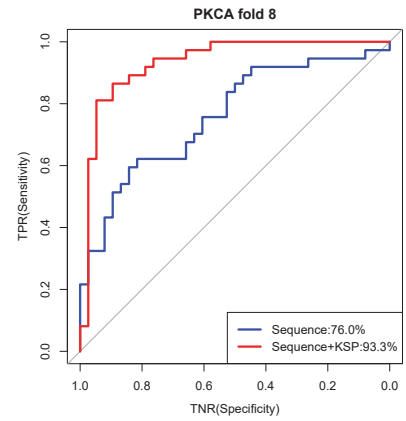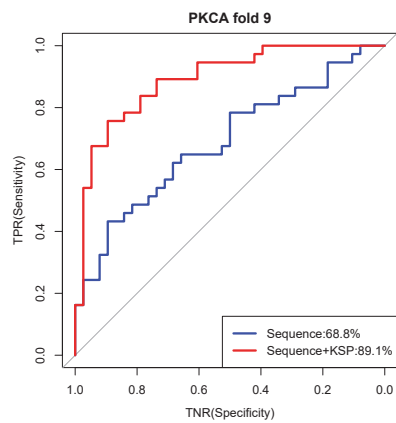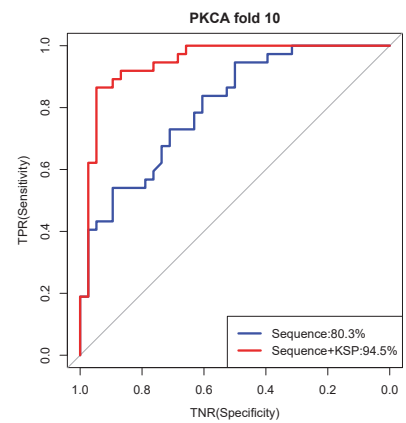

Figure S5. Results of the 10-fold cross validation on PKCA.

Supplement: Supplementary file 9 — Additional file 9: Figure S5. Results of the 10-fold cross validation experiment on PKCA. [file 12864_2020_6895_MOESM9_ESM.pdf]
